# Clinical and Sociodemographic Profile of Familial Amyotrophic Lateral Sclerosis Type 8 Compared to the Sporadic Form

**DOI:** 10.1055/s-0046-1817037

**Published:** 2026-03-23

**Authors:** Danilo Jorge da Silva, Sophia Calabria da Silveira, Leonardo Cruz de Souza, Marcelo Maroco Cruzeiro, Thiago Cardoso Vale

**Affiliations:** 1 Universidade Federal de Juiz de Fora, Faculdade de Medicina, Programa de Pós-Graduação em Saúde, Juiz de Fora MG, Brazil.; 2 Universidade Federal de Juiz de Fora, Faculdade de Medicina, Juiz de Fora MG, Brazil.; 3 Universidade Federal de Minas Gerais, Faculdade de Medicina, Belo Horizonte MG, Brazil.; 4 Universidade Federal de Juiz de Fora, Faculdade de Medicina, Departamento de Clínica Médica, Juiz de Fora MG, Brazil.; 5 Universidade Federal de Juiz de Fora, Hospital Universitário, Serviço de Neurologia, Juiz de Fora MG, Brazil.

**Keywords:** Amyotrophic Lateral Sclerosis, Motor Neuron Disease

## Abstract

**Background:**

Amyotrophic lateral sclerosis (ALS) is a rare degenerative disease of motor neurons, predominantly sporadic, with approximately 10% of the cases showing familial inheritance.

**Objective:**

To characterize the clinical and sociodemographic profile of patients with familial ALS type 8 (fALS8) and compare it with sporadic ALS (sALS).

**Methods:**

We reviewed the medical records (1997–2022) from a specialized Brazilian center. Patients with a confirmed diagnosis of ALSs were included, and sociodemographic and clinical data were collected.

**Results:**

The sample was composed of 89 ALS patients, with a slight female predominance (53%) and a high frequency of fALS8 cases (45%). The fALS8 patients were diagnosed at a younger age, at approximately 50 years, compared to 53 years among the sALS patients ( *p* = 0.043). Lower limb onset predominated in the fALS8 group (87%), while the sALS group showed more heterogeneous presentations, including bulbar onset (14%). The time until the diagnosis was significantly longer in the fALS8 group compared to the sALS group, both from symptom onset (approximately 51 versus 30 months respectively; *p* < 0.001) and after admission to a specialized center (7 versus 4 months respectively; *p* = 0.002). Dysphagia and gastrostomy were more frequent in the sALS group compared to the fALS8 group ( *p* = 0.02 and *p* < 0.01 respectively), and older age at diagnosis was associated with worse functional scores.

**Conclusion:**

The fALS8 group presented with distinct clinical and demographic features compared to the sALS group, including younger age at diagnosis, more homogeneous symptom onset, and lower frequency of dysphagia and need for gastrostomy. The diagnosis was more delayed in the fALS8 group, and older age at diagnosis was associated with worse functional status. The current study contributes to the scarce data on fALS8 in South America.

## INTRODUCTION

Amyotrophic lateral sclerosis (ALS) is a progressive and fatal motor neuron disease. It is the most common motor neuron disease, and it is most frequently observed between 58 and 63 years of age. 1 2 The clinical findings include symptoms compatible with the involvement of the upper and lower motor neurons, and most patients begin their symptoms asymmetrically in one limb or in both lower or upper limbs. These symptoms usually progress by contiguity through the medullary regions and are initially characterized by cramps, weakness, and fasciculations. 3 Bulbar symptoms onset is observed in approximately 30% of the cases. 4 Sporadic ALS (sALS) is a more common presentation, representing 80 to 90% of all patients, and most cases present initially with asymmetric appendicular weakness. In contrast, cases of familial ALS (fALS) account for roughly 10% of the total proportion of diagnoses, and they are associated with mutations in 20 to 30 already-established genes; these mutations are identifiable in 60 to 80% of the cases. 5 6

Familial ALS may be clinically defined as: having at least two first- or second-degree relatives with ALS or at least one relative with ALS and evidence of genetic inheritance. 7 8 Familial ALS type 8 (fALS8) is a fALS with an autosomal dominant nature associated with the p.P56S mutation in the vesicular membrane-associated protein ( *VAPB* ) gene, located on chromosome 20q13.3. 9 Its prevalence may represent more than 30% of the fALS cases in Brazil, 10 11 and it is rarely described in European populations.

The ALS phenotypes are markedly heterogeneous with respect to clinical course, age at onset, presence of associated extramotor features, and average survival. 9 11 The fALS8 patients present with a slower progression of symptoms when compared to sALS patients, and their symptoms commonly begin in the lower limbs with predominance of lower motor neuron involvement. 12 Autonomic dysfunction and mild cognitive dysfunction are also observed in ALS8. 13 14 15

There are few descriptions of ALS patient populations available in Brazil, and data from many referral centers are unavailable. In the state of Minas Gerais, for example, only data from the state capital are available. 16 The current study aims to characterize the clinical and sociodemographic profile of patients with fALS8e 8 and compare them to those with sALS at a specialized referral center in Minas Gerais, which is responsible for a large territory of 341 affiliated municipalities.

## METHODS

### Study design and sample

This is a descriptive, retrospective study based on secondary data. The information was obtained through the collection of physical and electronic medical records from consultations conducted at a specialized referral center, the Neuromuscular Disease Outpatient Clinic at the Teaching Hospital of Universidade Federal de Juiz de Fora (UFJF), between 1997 and 2022. The medical records were analyzed, and the signs and symptoms of ALS were classified and reviewed by two independent neurologists. Patients with clinically-definite ALS, according to the Revised El Escorial criteria, were included in the study. Cases with discrepancies in the ALS diagnosis were excluded. The individuals were classified as belonging to the fALS8 group if they had a compatible clinical presentation and molecular confirmation of a *VAPB* gene variant, or a compatible family history of one or more first- or second-degree relatives with a molecularly-confirmed diagnosis of fALS8, as suggested by Byrne et al 7 and Barberio et al. 8

### Clinical and sociodemographic data

The sociodemographic variables of interest collected were: age at the last outpatient visit, age at diagnosis, and sex. The clinical variables collected included: score on the Amyotrophic Lateral Sclerosis Functional Rating Scale–Revised (ALSFRS-R), 17 restriction to a wheelchair, complete bed restriction, presence of dysphagia, presence of gastrostomy, use of non-invasive ventilation through bilevel positive airway pressure (BiPAP) or advanced airway management (orotracheal intubation; OTI) or tracheostomy (TT).

Moreover, we analyzed the following data: total duration of the disease (the time from the first reported symptoms to the date of the last visit); the time elapsed between the date of the first reported symptoms and the diagnosis of ALS; and the time in months until diagnosis after admission to the medical reference service. We also established a correlation between this set of variables and the ALSFRS-R, and compared the fALS8 and sALS groups.

### Statistical analysis

The Shapiro-Wilk method was used to assess adherence to the normality assumption. The parametric continuous variables were expressed as mean ± standard deviation values, and the nonparametric variables were expressed as median and interquartile range (IQR) values. The categorical variables were expressed as absolute frequencies and percentages. The Student's *t* -test was used to compare the means, and the Mann‒Whitney U test, to assess differences in distribution between populations, based on the normality adjustment. The Chi-squared (χ ^2^ ) test or the Fisher's exact test was used as indicated for associations regarding variables. Correlations involving continuous variables were assessed using the Kendall's Tau-b method. The effect size was also estimated based on the Kendall's Tau-b, converting the Tau (τ) coefficient into the Pearson's correlation coefficient and, subsequently, into Cohen's *d* . 18 19

The cutoff points for categorization according to the magnitude of the effect size (based on the value of *d* ) followed Cohen's suggestions: 0.3 for small, 0.5 for moderate, and 0.8 for large effect sizes. 20 Statistical analyses were performed using the IBM SPSS Statistics for Windows (IBM Corp.) software, 26.0, assuming a critical *p* value of 5% for the rejection of the null hypotheses.

### Ethical considerations

The research project was approved by the Ethics in Research Committee of UFJF's Teaching Hospital (under CAAE number: 48912921.3.0000.5133).

## RESULTS

**Figure 1:**
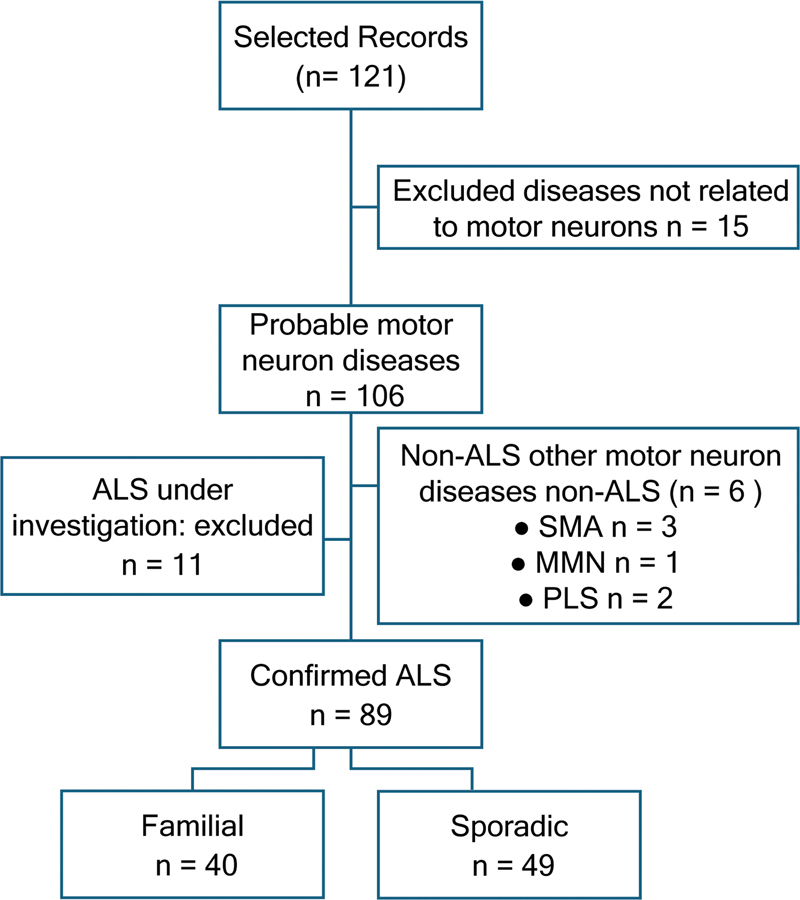
Abbreviations: ALS, amyotrophic lateral sclerosis; MMN, multifocal motor neuropathy; SMA, spinal muscular atrophy; PLS, primary lateral sclerosis. Study selection flowchart.

A total of 121 medical records were initially selected and evaluated, with 15 cases unrelated to motor neuron diseases being excluded. Of the remaining 106 cases of probable motor neuron disease, 11 were under diagnostic investigation for ALS, and 6 were excluded, as they were classified as other non-ALS motor neuron diseases (3 cases of spinal muscular atrophy, 1 case of multifocal motor neuropathy, and 2 cases of primary lateral sclerosis). In total, 89 patients were included in the study: 40 in the fALS8 group and 49 in the sALS group ( Figure 1 ). Genetic testing was only available for 15 (17%) of the fALS8 individuals.

Considering the whole sample, the mean age (at the last visit) was of 56.1 ± 9.0 years, and the mean age at diagnosis was of 52.9 ± 10.4 years, which was approximately 5 years younger in the fALS8 group ( *p* = 0.043). There was a slight predominance of female subjects, representing 52.8% of the total of cases (with this predominance observed only for the familiar cases when stratified). These data are summarized in Table 1 .

**Table 1:** Population profile

Variable		Total		sALS		fALS8		*p*
		N	Median (IQR)	N	Median (IQR)	N (%)	Median (IQR)	
Age at the last visit (years)		89	56.1 ± 9.0 a	49	57.9 ± 9.6 a	40	53.9 ± 7.8 a	0.039
Age at diagnosis (years)		87	52.9 ± 10.4 a	48	55.0 ± 10.2 a	39	50.4 ± 10.3 a	0.043
Sex: n (%)	Male	42 (47.2%)		26 (53.1%)		16 (40.0%)		0.220
	Female	47 (52.8%)		23 (46.9%)		24 (60.0%)		
Total duration of the disease (months) *		88	68.3 (88.0)	48	38.2 (62.5)	40	107.7 (90.2)	< 0.001
Symptom-to-diagnosis time (months) **		88	30.3 (38.4)	49	20.7 (21.4)	39	51.3 (72.6)	< 0.001
Time until diagnosis after admission to the medical reference service (months)		69	4.4 (15.3)	48	1.3 (4.9)	38	6.9 (24.0)	0.002

Abbreviations: fALS8, familial amyotrophic lateral sclerosis type 8; IQR, interquartile range; sALS, sporadic amyotrophic lateral sclerosis.

Notes: ^a^ Data for age are expressed in mean ± standard deviation values; *time from the first reported symptoms to the date of the last visit; **time from the date of the first reported symptoms to the diagnosis of ALS.

The median value for the total duration of the disease was of 68.3 (IQR: 88.0) months, and the median symptom-to-diagnosis time was of 30.3 (IQR: 38.4) months for the whole sample. The median time until diagnosis after admission to the medical reference service was of 4.4 (IQR: 15.3) months, with 18 patients excluded from this analysis because of a complete diagnosis on admission to the service, and 2 patients with incomplete information. The time until diagnosis was significantly longer in the fALS8 group, with 6.9 against 1.3 months in the sALS group ( *p* = 0.002), and the cumulative duration of the disease was approximately 2.5 times longer in the fALS8 group ( *p* < 0.001). The median time from the first symptoms until diagnosis was of 20.7 months for the sALS group and of 51.3 months for the fALS8 group.

The total sample presented a greater prevalence of spinal manifestations, with 54 (60.7%) patients exhibiting symptoms starting in the lower limbs, and 16 (18.0%), in the upper limbs. Notably, the initial onset in the lower limbs was more frequent in those with fALS8, occurring in 35 (87.5%) patients. Bulbar presentations comprised 7 (14.3%) of the sALS cases, whereas none was observed among patients with fALS8. The frequencies of other less common presentations are summarized in Table 2 .

**Table 2:** Initial ALS phenotypes observed according to form

	Total		sALS		fALS8	
Initial phenotype	No.	%	No.	%	No.	%
Spinal	82	92.1	42	85.7	40	100
Spinal – LL	54	60.7	19	38.8	35	87.5
Spinal – UL	16	18.0	14	28.6	2	5.0
Pseudopolyneuritic	5	5.6	3	6.1	2	5.0
Spinal – UL and LL	2	2.2	1	2.0	1	2.5
Flail legs	2	2.2	2	4.1	0	–
Mills	2	2.2	2	4.1	0	–
Flail arms	1	1.1	1	2.0	0	–
Bulbar	7	7.9	7	14.3	0	–
Total	89	100	49	100	40	100

Abbreviations: ALS, amyotrophic lateral sclerosis; fALS8, familial amyotrophic lateral sclerosis type 8; LL, lower limbs; Mills, Mills hemiplegic variant; sALS, sporadic amyotrophic lateral sclerosis; UL, upper limbs.

The survey of clinical landmarks of interest described the need to use BiPAP in 36 (42.9%) of the patients in the total sample with valid data, and cases of tracheostomy were described in 6 (7.1%) individuals. Dysphagia was reported in 50 (56.2%) subjects, and it was significantly more prevalent in the sALS group (67.3%) compared to the fALS8 group (42.5%). The odds of an individual with sALS having dysphagia were 2.79 times greater when compared to fALS8 individuals (χ ^2^ *p* = 0.02; 95%CI 1.17–6.62). No patient with fALS underwent gastrostomy, which was performed in 11 (22.4%) patients with sALS (Fisher's exact test *p* < 0.01). Regarding motor function, 38 (42.7%) subjects were restricted to a wheelchair, and 3 (3.4%) were bedridden. The clinical landmark differences between the fALS8 and sALS groups are shown in Table 3 .

**Table 3:** Clinical landmarks of interest according to ALS form

	sALS		fALS8		*p* *
	N	n (%)	N	n (%)	
BiPAP	46	21 (45.7)	38	15 (39.5)	0.57
OTI/TQT	47	2 (4.3)	37	4 (10.8)	0.25
Dysphagia	49	33 (67.3)	40	17 (42.5)	0.02
Gastrostomy	49	11 (22.4)	40	0 (0.0)	< 0.01
Restricted to a wheelchair	49	22 (44.9)	40	16 (40.0)	0.64
Restricted to bed (bedridden)	49	2 (4.1)	40	1 (2.5)	0.68

Abbreviations: ALS, amyotrophic lateral sclerosis; BIPAP, bilevel positive airway pressure; fALS8, familial amyotrophic lateral sclerosis type 8; OTI, orotracheal intubation; sALS, sporadic amyotrophic lateral sclerosis; TQT, tracheostomy.

Note: *Chi-squared test or Fisher's exact test.

Functional evaluations using the ALSFRS-R were found in 23 medical reports (6 for sALS and 17 for fALS8). The mean ALSFRS-R score (at the last visit) was of 30.2 ± 11.0 points, and no significant differences were found between the fALS8 and sALS groups; these patients had mean scores of 31.8 ± 8.3 and 25.7 ± 16.9 points respectively ( *p* = 0.428). There was a significant negative correlation with large effect size regarding the ALSFRS-R score and the current age (Kendall's Tau-b = −0.37; *d* = 1.3; *p* = 0.017) and the age at diagnosis (Kendall's Tau-b = −0.32; *d* = 1.1; *p* = 0.042). The total duration of the disease also showed a significant negative association with the ALSFRS-R score (Kendall's Tau-b = −0.33; *d* = 1.1; *p* = 0.030), as shown in Table 4 .

**Table 4:** ALSFRS-R scores according to the variables of interest

Variable		n	ALSFRS-R		*p* *
			Mean	σ	
Form	sALS	6	25.7	16.9	0.428
	fALS8	17	31.8	8.26	
		**n**	**τ**	**Cohen's** ***d***	*p* **
Current age (years)		23	−0.369	1.309	0.017
Age at diagnosis (years)		23	−0.315	1.079	0.042
Schooling		16	−0.027	0.085	0.890
Total duration of the disease		23	−0.330	1.141	0.030
Time until diagnosis after admission		18	0.128	0.408	0.469
Symptom-to-diagnosis time		23	−0.113	0.359	0.458

Abbreviations: ALSFRS-R, Amyotrophic Lateral Sclerosis Functional Rating Scale–Revised; fALS8, familial amyotrophic lateral sclerosis type 8; sALS, sporadic amyotrophic lateral sclerosis.

Notes: *Student's *t* -test; **Kendall's Tau-b.

## DISCUSSION

In the current study, we compared the clinical and demographic profiles of patients with sALS and fALS8 and found significant differences between the groups regarding diagnostic trajectory, initial phenotype, and clinical landmarks of interest.

The extremely high prevalence of fALS8 linked to the *VAPB* gene in our sample highlights the proximity of our center to a population cluster of this form of ALS. Familial ALS8 was originally described in the vicinity of our reference center, in the city of Guarani, in 1962. 21 A common founder gene is linked to Portugal and was carried to Brazil during the colonial period, characterizing a new founding event, 22 and cases observed outside of this particular area are rare.

The obtained sample is in accordance with the literature regarding the mean age at disease onset. However, an unusually-small predominance of female patients was observed. Although the proportions of male and female subjects vary widely among studies, with a male-to-female ratio typically between 1 and 2, 23 the observation of a female predominance is not a common phenomenon, and it may be explained by the presence of a significant fraction of the study population represented by patients with fALS8. A limitation in the evaluation of the fALS8 population is that the data available in the literature do not come from large studies, and there is a significant shortage of epidemiological information from this group of patients. Smaller-scale studies 9 14 suggest distinct epidemiology, with equivalent incidences between genders or attenuated male predominance for fALS8, with a recent study 24 implying a marginal predominance of female patients for this subpopulation based on a small series of five cases.

The phenotypic distribution deserves consideration. The number of patients with bulbar onset was slightly lower than anticipated, in contrast to data from the current literature, 4 23 25 which reports rates ranging from 19.0 to 35.0% in different samples. However, the absence of cases with bulbar onset among patients with fALS8 and the predominance of phenotypes with symptoms beginning in the lower limbs in this population are in agreement with the case series described in the literature to date. 4 9 11

Despite the severity of the disease, delayed diagnosis is still common in ALS patients, 4 and the mean time until diagnosis varies among different studies in different locations and services. This delay is in part due to the lack of a definitive biomarker for ALS, making it necessary to rule out other diseases during the diagnostic process. Despite the previous remark, issues related to the Brazilian public healthcare system may significantly contribute to diagnostic delay, as discussed in previous Brazilian studies. 16 Among these issues are difficulties in accessing complementary diagnostic tools and possible delays in referring patients to neurologists or specialized centers. The time spent for diagnosis varies from 17 to 21 months, as described in a recent multicenter retrospective study 26 of 201 patients in 6 countries (Argentina, Brazil, Germany, Italy, Spain, and the United States), and it depends on how many medical specialties evaluated the patient before the first appointment with the neurologist. In our combined cohort, the diagnostic delay was longer than previously reported, measured from the onset of ALS symptoms, with considerable variability in the time required to reach a definitive diagnosis. This phenomenon may be explained by the predominance of fALS8 patients in the sample, who tended to experience longer diagnostic delays, whereas the time until diagnosis for sALS patients was in line with literature data.

The same multicenter study 26 reported that an average of 7 months is expected for diagnostic confirmation after admission to neurology services, while another cohort of 304 patients in the United States 27 found that the mean time from symptom onset until diagnosis was of 11.5 months, with a median of 4 months after the initiation of the investigation due to a suspected diagnosis. The delay in accessing specialized services is probably the main factor responsible for the longer overall diagnostic time, since once patients were admitted to our referral center, the time until diagnosis was relatively short for both groups. These findings indicate that, although the median diagnostic time for sALS was shorter than commonly reported, the fALS8 patients still faced a considerably longer interval until diagnosis, even after admission to specialized care.

A possible explanation for the significantly longer time until diagnosis of fALS in our sample is the atypical presentation of fALS8 and its more indolent course, with a tendency to maintain symptoms exclusive to the lower motor neuron for longer periods until the first signs related to the upper motor neuron can be identified. 9 There is also agreement with the literature 26 28 suggesting that the onset of symptoms in the lower limbs (which was observed in a large proportion of our sample) tends to prolong the total time until diagnosis, while bulbar-onset symptoms typically result in an earlier diagnosis of ALS. The absence of typical signs such as hyperreflexia, and the presence of uncommon phenomena such as postural or other forms of tremor, 9 29 can contribute to difficulties in establishing a diagnosis.

The higher proportion of patients undergoing gastrostomy and with dysphagia among those with sALS can be explained by the proximity of the bulbar region to the primary site of degeneration in the cervical spinal cord. In contrast, the slow progression of initial symptoms in fALS8 patients, starting in the lumbar spinal cord, means bulbar symptoms typically appear only in the advanced stages of fALS8. However, the absence of significant differences between the ALSFRS-R score and BiPAP between patients with sALS and fALS8 should be analyzed with caution, since a limitation of the current study was the impossibility of weighting the ALSFRS-R scores according to the need for ventilatory support for each subgroup, given the limitations in the availability of data from the functional assessments.

As expected, greater functional impairments measured by the ALSFRS-R were associated with the use of BiPAP, the presence of dysphagia, and inability to walk, and a higher number of sALS patients required some form of ventilatory support, were under gastrostomy, and presented with dysphagia compared to the fALS8 patients in our sample. Worse functional performance was also associated with older age at diagnosis and longer total disease duration, which is consistent with findings in the literature. 30 31 The less severe clinical profile of fALS8, evidenced by the reduced need for supportive interventions, may contribute to the extended survival observed in this group. This observation is consistent with previous descriptions of a slower functional decline in fALS8.

The data collected suggest that recognizing population patterns and their genetic profiles may help shorten the time until diagnosis and, consequently, enable earlier intervention, since clinical presentation, progression, and prognosis can vary significantly among different ALS forms. Moreover, failure to recognize atypical presentations of ALS, beyond the typical sporadic form, may deny patients the necessary support. In addition to the possible difficulties in observing the first symptoms at an early stage of the disease (especially in milder phenotypes), the many consultations with different non-neurologist professionals can be a source of delay in diagnosis.

Unfortunately, data related to the patient's consultations with different professionals until the arrival at our specialized center could not be evaluated in the present study. The study also has limitations inherent to its retrospective design, with secondary data collection, for which it was not possible to retrieve sufficient ALSFRS-R data to stratify the results according to fALS8 and sALS. This limitation was compounded by the scarcity of functional data, since ALSFRS-R scores were only available for a small number of subjects, substantially limiting the strength of the functional comparisons. While the ALSFRS-R scores did not differ significantly between the groups, the small sample size—particularly in the sALS cohort—restricts the strength of these findings, and the absence of significant differences should be interpreted with caution. Another limitation worthy of attention is that not all patients with fALS had access to molecular testing. Although presumed cases were defined conservatively, requiring a compatible clinical picture plus at least one first- or second-degree relative with genetically-confirmed ALS8, the possibility of misclassification cannot be entirely excluded. This issue reflects the absence of standardized international criteria for FALS, 7 despite commonly-used definitions based on family history 8 .

The present study compiles a set of clinical and sociodemographic characteristics of patients with sALS and fALS8. The fALS8 group was diagnosed at younger ages and had a slight overall predominance of females, which was not observed in the sALS group. Clinically, the fALS8 group had a distinct phenotype, characterized by a more homogeneous onset of symptoms and absence of initial bulbar presentations. Additionally, both the need for gastrostomy and the presence of dysphagia were observed less frequently in the fALS8 group.

As one of the main points observed, the data shows a significantly longer diagnostic delay inthe fALS8 group, from symptom onset and after admission to a reference neurology service. Despite the mild presentation of fALS8, worse functional scores were significantly associated with older age at diagnosis and longer disease duration, highlighting the importance of early detection and clinical support for the condition for all ALS patients.

The results highlight the clinical heterogeneity between fALS8 and sALS, and they underscore the importance of recognizing distinct phenotypic patterns to improve diagnostic efficiency and clinical support. Although the data collection method used has limitations—such as the inability to recover the temporal evolution of ALSFRS-R scores—it provides a valuable overview of a large ALS population with a significant number of fALS8 cases. This contributes to the limited population-level data on ALS in South America, particularly regarding fALS8.

## References

[1] Chiò A Logroscino G Traynor B J Global epidemiology of amyotrophic lateral sclerosis: a systematic review of the published literature Neuroepidemiology 2013 41 02 118 130 10.1159/000351153 23860588 PMC4049265

[2] Connolly O Le Gall L McCluskey G Donaghy C G Duddy W J Duguez S A systematic review of genotype–phenotype correlation across cohorts having causal mutations of different genes in ALS J Pers Med 2020 10 03 58 10.3390/jpm10030058 32610599 PMC7564886

[3] Talbott E O Malek A M Lacomis D The epidemiology of amyotrophic lateral sclerosis Handb Clin Neurol 2016 138 225 238 10.1016/B978-0-12-802973-2.00013-6 27637961

[4] Hardiman O Van den Berg L H Kiernan M C Clinical diagnosis and management of amyotrophic lateral sclerosis Nat Rev Neurol 2011 7 11 639 649 10.1038/nrneurol.2011.153 21989247

[5] Al-Chalabi A Hardiman O Kiernan M C Chiò A Rix-Brooks B Van den Berg L H Amyotrophic lateral sclerosis: moving towards a new classification system Lancet Neurol 2016 15 11 1182 1194 10.1016/S1474-4422(16)30199-5 27647646

[6] Corcia P Couratier P Blasco H Genetics of amyotrophic lateral sclerosis Rev Neurol (Paris) 2017 173 05 254 262 10.1016/j.neurol.2017.03.030 28449881

[7] Byrne S Bede P Elamin M Proposed criteria for familial amyotrophic lateral sclerosis Amyotroph Lateral Scler 2011 12 03 157 159 10.3109/17482968.2010.545420 21208036

[8] Barberio J Lally C Kupelian V Hardiman O Flanders W D Estimated Familial Amyotrophic Lateral Sclerosis Proportion: A Literature Review and Meta-analysis Neurol Genet 2023 9 06 e200109 10.1212/NXG.0000000000200109 38045991 PMC10689005

[9] Nishimura A L Mitne-Neto M Silva H CA A mutation in the vesicle-trafficking protein VAPB causes late-onset spinal muscular atrophy and amyotrophic lateral sclerosis Am J Hum Genet 2004 75 05 822 831 10.1086/425287 15372378 PMC1182111

[10] Chadi G Maximino J R Jorge F MH Genetic analysis of patients with familial and sporadic amyotrophic lateral sclerosis in a Brazilian Research Center Amyotroph Lateral Scler Frontotemporal Degener 2017 18(3-4):249 255 10.1080/21678421.2016.1254245 27978769

[11] Gonçalves J PN Leoni T B Martins M P Genetic epidemiology of familial ALS in Brazil Neurobiol Aging 2021 102 2270 2.27E6 10.1016/j.neurobiolaging.2021.01.007 33618928

[12] Kosac V Freitas M R Prado F M Nascimento O JM Bittar C Familial adult spinal muscular atrophy associated with the VAPB gene: report of 42 cases in Brazil Arq Neuropsiquiatr 2013 71 10 788 790 10.1590/0004-282X20130123 24212516

[13] Alcântara Cd Cruzeiro M M França M C Jr A comparative study of cognitive and behavioral profiles between sporadic and type 8 amyotrophic lateral sclerosis Muscle Nerve 2023 68 03 316 322 10.1002/mus.27927 37424512

[14] Alcântara Cd Cruzeiro M M França M C Jr Camargos S T Souza LCd Amyotrophic lateral sclerosis type 8 is not a pure motor disease: evidence from a neuropsychological and behavioural study J Neurol 2019 266 08 1980 1987 10.1007/s00415-019-09369-y 31089860

[15] Marques V D Barreira A A Davis M B Expanding the phenotypes of the Pro56Ser VAPB mutation: proximal SMA with dysautonomia Muscle Nerve 2006 34 06 731 739 10.1002/mus.20657 16967488

[16] Prado LdGR Bicalho I CS Vidigal-Lopes M Amyotrophic lateral sclerosis in Brazil: Case series and review of the Brazilian literature Amyotroph Lateral Scler Frontotemporal Degener 2016 17(3-4):282 288 10.3109/21678421.2016.1143011 26854959

[17] BDNF ALS Study Group (Phase III) Cedarbaum J M Stambler N Malta E The ALSFRS-R: a revised ALS functional rating scale that incorporates assessments of respiratory function J Neurol Sci 1999 169(1-2):13 21 10.1016/s0022510x(99)00210-5 10540002

[18] Walker D A JMASM9: Converting Kendall's Tau For Correlational Or Meta-Analytic Analyses J Mod Appl Stat Methods 2003 2 02 525 530 10.22237/jmasm/1067646360

[19] Valentine J C Hedges L V Cooper H M The handbook of research synthesis and meta-analysis New York Russell Sage Foundation 2009

[20] Cohen J Statistical Power Analysis for the Behavioral Sciences 2nd Eed. Mahwah, NJ Erlbaum Associates 1988. Available from:https://utstat.utoronto.ca/brunner/oldclass/378f16/readings/CohenPower.pdf

[21] Finkel N A forma pseudomiopática tardia da atrofia muscular progressiva heredo-familial Arq Neuro-Psiquiatr 1962 20 04 307 322 10.1590/S0004-282X1962000400005

[22] Nishimura A L Al-Chalabi A Zatz M A common founder for amyotrophic lateral sclerosis type 8 (ALS8) in the Brazilian population Hum Genet 2005 118(3-4):499 500 10.1007/s00439-005-0031-y 16187141

[23] Longinetti E Fang F Epidemiology of amyotrophic lateral sclerosis: an update of recent literature Curr Opin Neurol 2019 32 05 771 776 10.1097/WCO.0000000000000730 31361627 PMC6735526

[24] Trilico M LC Lorenzoni P J Kay C SK Characterization of the amyotrophic lateral sclerosis-linked P56S mutation of the *VAPB* gene in Southern Brazil Amyotroph Lateral Scler Frontotemporal Degener 2020 21(3-4):286 290 10.1080/21678421.2020.1738495 32162544

[25] Turner M R Scaber J Goodfellow J A Lord M E Marsden R Talbot K The diagnostic pathway and prognosis in bulbar-onset amyotrophic lateral sclerosis J Neurol Sci 2010 294(1-2):81 85 10.1016/j.jns.2010.03.028 20452624

[26] Chiò A ISIS Survey: an international study on the diagnostic process and its implications in amyotrophic lateral sclerosis J Neurol 1999 246 03 III1 III5 10.1007/BF03161081 10631652

[27] Paganoni S Macklin E A Lee A Diagnostic timelines and delays in diagnosing amyotrophic lateral sclerosis (ALS) Amyotroph Lateral Scler Frontotemporal Degener 2014 15(5-6):453 456 10.3109/21678421.2014.903974 24981792 PMC4433003

[28] Nzwalo H Abreu Dd Swash M Pinto S Carvalho Md Delayed diagnosis in ALS: the problem continues J Neurol Sci 2014 343(1-2):173 175 10.1016/j.jns.2014.06.003 24972820

[29] Di L Chen H Da Y Wang S Shen X M Atypical familial amyotrophic lateral sclerosis with initial symptoms of pain or tremor in a Chinese family harboring VAPB-P56S mutation J Neurol 2016 263 02 263 268 10.1007/s00415-015-7965-3 26566915

[30] Eurals Consortium Chiò A Logroscino G Hardiman O Prognostic factors in ALS: A critical review Amyotroph Lateral Scler 2009 10(5-6):310 323 10.3109/17482960802566824 19922118 PMC3515205

[31] Su W M Cheng Y F Jiang Z Predictors of survival in patients with amyotrophic lateral sclerosis: A large meta-analysis EBioMedicine 2021 74 103732 10.1016/j.ebiom.2021.103732 34864363 PMC8646173

